# Electrodeposition of (hydro)oxides for an oxygen evolution electrode

**DOI:** 10.1039/d0sc01532f

**Published:** 2020-04-20

**Authors:** Zhenhua Yan, Huanhuan Liu, Zhimeng Hao, Meng Yu, Xiang Chen, Jun Chen

**Affiliations:** Key Laboratory of Advanced Energy Materials Chemistry (Ministry of Education), Renewable Energy Conversion and Storage Center, College of Chemistry, Nankai University Tianjin 300071 China chenabc@nankai.edu.cn

## Abstract

Electrochemical water splitting is a promising technology for hydrogen production and sustainable energy conversion, but the electrolyzers that are currently available do not have anodic electrodes that are robust enough and highly active for the oxygen evolution reaction (OER). Electrodeposition provides a feasible route for preparing freestanding OER electrodes with high active site utilization, fast mass transport and a simple fabrication process, which is highly attractive from both academic and commercial points of view. This minireview focuses on the recent electrodeposition strategies for metal (hydro)oxide design and water oxidation applications. First, the intrinsic advantages of electrodeposition in comparison with traditional technologies are introduced. Then, the unique properties and underlying principles of electrodeposited metal (hydro)oxides in the OER are unveiled. In parallel, illustrative examples of the latest advances in materials structural design, controllable synthesis, and mechanism understanding through the electrochemical synthesis of (hydro)oxides are presented. Finally, the latest representative OER mechanism and electrodeposition routes for OER catalysts are briefly overviewed. Such observations provide new insights into freestanding (hydro)oxides electrodes prepared *via* electrodeposition, which show significant practical application potential in water splitting devices. We hope that this review will provide inspiration for researchers and stimulate the development of water splitting technology.

## Abstract 2

This minireview looks at recent electrodeposition strategies for metal (hydro)oxide design and water oxidation applications, unveiling the unique properties and underlying principles of electrodeposited metal (hydro)oxides in the OER.

## Graphical abstract

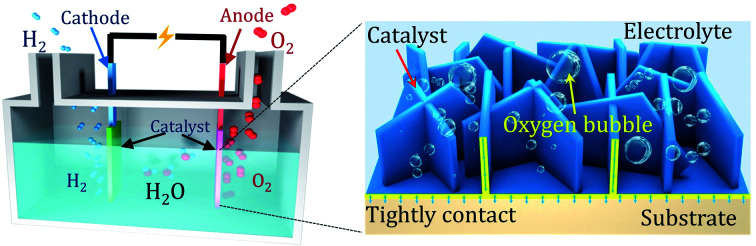

## Introduction

**Fig. 1:**
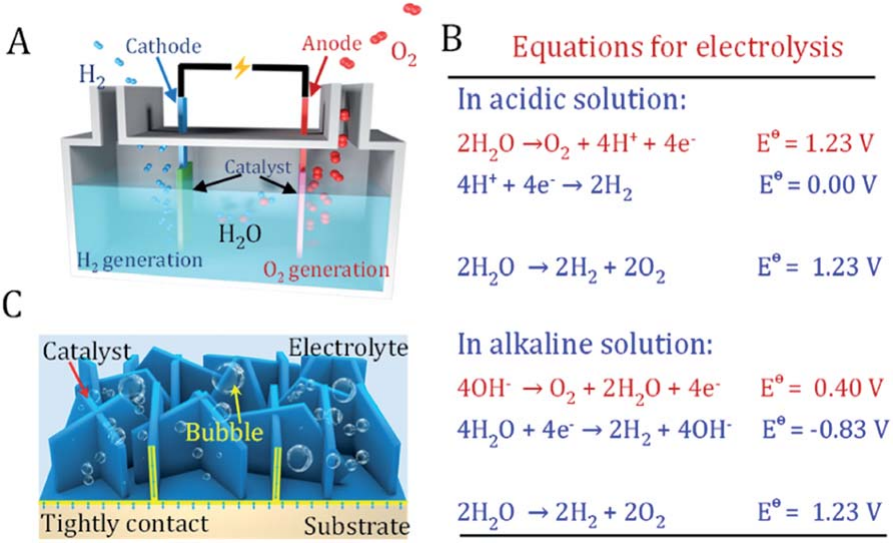
Principles of water splitting devices. (A) Schematic illustration of the water splitting cell. (B) The reactions at the anode and cathode under the acidic and alkaline conditions of water splitting. (C) The freestanding oxygen evolution reaction electrode prepared *via* electrodeposition has close contact between the catalyst and substrate, avoiding the use of conducting carbon and polymeric binder.

As an ideal energy carrier with an ultrahigh caloric value and CO_2_-free emissions, hydrogen is considered as the ultimate chemical energy source.^1–3^ Electrochemical splitting of water into hydrogen and oxygen provides a promising route to produce hydrogen. Simultaneously, it stores intermittent energies such as solar and wind in the form of chemical energy (Fig. 1A).^4^ This green route allows the production of high-level hydrogen with almost zero carbon emissions. However, the anodic four-electron transfer oxygen evolution reaction (OER) in water splitting typically requires a larger overpotential than the cathodic two-electron hydrogen evolution reaction (HER) (Fig. 1B).^5^ The equilibrium potential of oxygen evolution is as high as 1.23 V *vs.* RHE. Under OER conditions, conductive carbon and most materials are prone to be oxidized and etched at a high overpotential, resulting in the strong deterioration of electrode performance.^6^ Another difficulty is that rigorous bubble release under high current density will inevitably cause serious bubble-shielding effects and catalyst peel off issues.^7,8^ Therefore, the OER catalyst and electrodes are the bottleneck in water splitting devices.

The successful utilization of water splitting technology heavily depends on exploring novel OER electrocatalytic materials and robust electrodes. A good OER electrode should not only have a highly active catalyst well assembled onto its surface, but should also have the features of an open structure. This allows rapid mass transfer of electrolyte and bubbles as well as strong adhesion between the catalyst and substrate (Fig. 1C).^6,9,10^ Furthermore, a facile and scalable fabrication procedure of an active electrode with affordable equipment is also highly favorable to realize mass production.

Owing to their special electronic structure, stable chemical properties, and high intrinsic activity, transition metal (hydro)oxides are promising OER electrocatalysts.^11–13^ To prepare metal (hydro)oxide-based electrodes, typical synthetic strategies, including hydro/solvothermal, vapor deposition, high-temperature solid-state reaction, and sol–gel methods have been developed.^14–17^ Most products are in powder form and need to be mixed with conducting carbon and polymeric binder into a slurry for further use. Such routes, which are relatively complex, have low utilization of active sites and easily result in catalyst peel off issues. In contrast, electrodeposition is a unique technology to prepare electrode materials that has the following advantages.^18–21^ First, electrochemical synthesis takes place inside the nanoscale thickness of the electric double layer with a high potential gradient of up to 10^5^ V cm^−1^.^18^ Many materials that are difficult to obtain *via* chemical methods under ambient conditions can be synthesized. Second, electrodeposition is mainly a surface-induced reaction, and available for interface engineering, which will be particularly beneficial for electrocatalysis OER applications. Third, self-standing electrodes can be easily fabricated *via* electrodeposition with the deposits rigidly attached to substrate. Electrochemically synthesized freestanding electrodes have the great advantages of high active site utilization and simpler manufacture processes than conventional drop-casted electrodes.^22,23^ Fourth, the electrode composition can be readily adjusted by varying the types of precursor solutions to fabricate almost all metal-based materials. Finally, electrodeposition is a low-cost solution-based method that is operable under ambient conditions, which is suitable for industrial amplification in practice. Benefiting from the above advantages, many of the recent innovations in water splitting have been achieved through electrodeposition, which has generated unprecedented interest.^24–27^

Although some excellent review articles on electrodeposition have been published, they mainly focus on alloys, batteries, or photoelectrodes.^18,21,28^ The latest progress on metal (hydro)oxide OER electrocatalysts prepared *via* electrodeposition has rarely been reviewed. In this minireview, we focus on electrodeposition strategies for metal (hydro)oxide design and OER applications. First, we summarize the properties of metal (hydro)oxides for the OER. Then, the fundamental principles of electrodeposition strategy and nanostructured electrodes fabrication are introduced. At last, we discuss the latest proposed OER mechanisms and applications of the resulting (hydro)oxide electrocatalyst materials in water splitting.

### Metal (hydro)oxide structures and properties

**Fig. 2:**
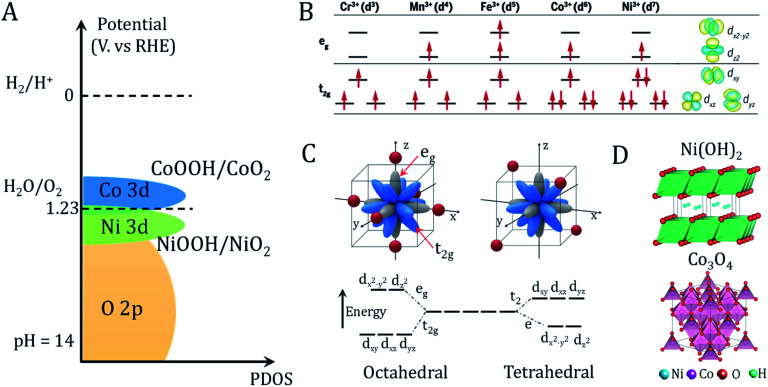
Redox properties and electronic structures of metal oxides/hydroxides. (A) Schematic of the valence band alignment of oxyhydroxides involving NiOOH/NiO_2_ and CoOOH/CoO_2_. Adapted with permission from ref. 11. Copyright 2018, Elsevier Inc. (B) Electronic configuration and relevant metal orbitals of first-row transition metals. Adapted with permission from ref. 30. Copyright 2017, AAAS (C) orbital splitting of transition metal in octahedral (six-oxygen coordinated environment) and tetrahedral (four-oxygen coordinated environment) geometries. Adapted with permission from ref. 31. Copyright 2019, John Wiley & Sons, Inc. (D) Crystal structure of representative hydroxide (Ni(OH)_2_) and spinel oxide (Co_3_O_4_).

The large family of transition metal (TM) (hydro)oxides are widely studied due to their low cost and promising OER activity. In metal (hydro)oxides, the hybridization of the metal d orbitals and ligand orbitals such as O 2p occur due to the spatial overlap and energetic similarity of the electronic states (Fig. 2A).^11^ The redox potential position, which reflects the electronic structure and surface oxygen adsorption strength, can be used as an effective descriptor for the OER activity of metal (hydro)oxide catalysts.^11^ Shifting the redox potential positively *via* the inductive effect of foreign metal ions could thus lead to higher catalytic activity. The occupation and species of TM cations at octahedral and tetrahedral sites are important variables in controlling the electronic structure.^29^

As shown in Fig. 2B, e_g_ state filling depends on the number and spin state of the d electrons. Compared to the t_2g_ states, the absorbed reaction intermediates tend to interact with the vertically oriented e_g_ orbitals of the metal ions.^30^ These interactions determine the energy gained by adsorption and desorption of adsorbates on the metal ions. It is noted that the TM ions present different coordination environments in octahedral and tetrahedral coordinated crystal fields. Octahedrally coordinated TM orbitals directly point toward the six adjacent ligands oxygen atoms and generate great orbital overlap (Fig. 2C).^29,31^ In contrast, the t_2_ or e orbitals of tetrahedrally coordinated TMs have less orbital overlap with the oxygen. Because the t_2_ or e orbitals point in directions that deviate significantly from the four adjacent oxygens, the TM–O hybridization orbital state in the metal (hydro)oxide will affect the covalency, bond arrangement and bond angles of the TM–O. This will affect the adsorption free energy of oxygen-containing intermediates, which has been applied to interpret the geometric effects on the OER activity.^12^ The two basic crystal structures of the most studied (hydro)oxides, Ni(OH)_2_ and Co_3_O_4_, are presented in Fig. 2D. Many advanced bimetallic (hydro)oxide OER electrocatalysts based on Ni(OH)_2_ and Co_3_O_4_ have been explored *via* electrodeposition.^32–35^ In this minireview, we focus on the recent discoveries of electrode preparation and OER applications of (hydro)oxides using the electrodeposition technique.

### Electrodeposition

Electrodeposition is an established technique with a history of more than two centuries. In 1800, Volta developed the first voltaic pile, successfully converting chemical energy into electricity and making continuous electricity available.^36^ In 1807, Davy first precisely used electricity power to discover new elements. The alkali metals sodium and potassium were the earliest products obtained *via* electrosynthesis.^37^ Since then, the scientific community has witnessed the rapid development of electrodeposition technology and the underlying mechanisms. Nowadays, electrodeposition has been accepted as one of the most ideal methods to produce OER electrocatalytic materials. These electrocatalytic materials usually have adjustable shape, thickness, or dimensions.^21,24,38–40^

**Fig. 3:**
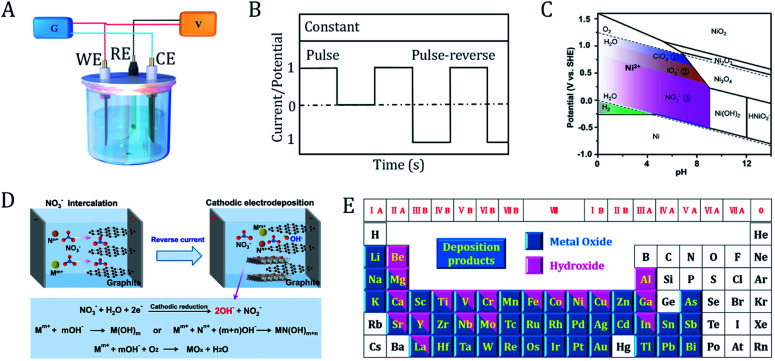
Principles of the electrodeposition of (hydro)oxides. (A) Electrolytic cell for electrodeposition. G, galvanostat; V, voltmeter; WE, working electrode; RE, reference electrode; CE, counter electrode. (B) Current/potential-controlled electrodeposition. (C) Pourbaix diagram of Ni-based species. The colored regions correspond to the oxoanion reactions that can be applied for the synthesis of nickel hydroxides. (D) Schematic illustration of the reaction mechanisms of the cathodic electrodeposition of metal (hydro)oxides, as well as the interface engineering process. M^*m*+^ and N^*n*+^ are metal cations. (C) and (D) are adapted from ref. 8. Copyright 2018, with permission from Nature Publishing Group. (E) Metal oxides and hydroxides that can be prepared *via* electrodeposition.

The principles of electrodeposition are shown in Fig. 3A. First, the reactants are dissolved in the electrolyte. By fine-tuning the applied cell potential, the oxidized or reduced products can be continuously deposited on the surface of the working electrode or counter electrode. A reference electrode is used to monitor the potential at the working electrode. Two important parameters that determine the course of the reaction are the deposition current and cell potential, which can be controlled as a function of time during the reaction. The size and morphology of the products can be tuned by optimizing various electrodeposition parameters (*e.g.*, time, current, potential, additives, pH and temperature).

In a current controlled synthesis process (Fig. 3B), the nucleation rate of the crystal can be tuned by accurately selecting a current and time to obtain deposits with a controlled morphology and good adhesion. In a potential controlled synthesis, the potential to be employed is usually not clear beforehand. Therefore, broad cell potential linear voltammetry is necessary to determine a suitable deposition potential. The deposition potential commonly lies within the voltage range of electrolyte decomposition. For aqueous electrolytes, potentiostatic synthesis can be carried out between the hydrogen evolution and oxygen evolution potentials. If the reaction potential falls outside the window of the electrolyte, we should choose a solvent (such as ionic liquids, organic solvents, and molten salts) with a wide potential window. In the case of constant potential, the cell current usually decays rapidly due to the low diffusion rate of the reactant molecules from the bulk solution to the electrode surface. This can be alleviated by stirring the electrolyte, rolling the electrode or changing the deposition mode to pulse potential.

### Electrodeposition mechanism of (hydro)oxides

Most metals can be electrodeposited directly at the cathode, while metal oxides tend to be produced at the anode when an electric current passes through a metal-salt solution. In electrochemical oxidation, a metal ion in a lower oxidation state can be oxidized to a higher oxidation state at the anode. The higher oxidation state readily undergoes hydrolysis to yield the metal oxide or hydroxide:M^*n*+^ → M^(*n*+*α*)+^ + *α*e^−^M^(*n*+*α*)+^ + (*n* + *α*)OH^−^ → M(OH)_*n*+*α*_ → MO_(*n*+*α*)/2_ + (*n* + *α*)/2 H_2_O

These routes have been used to synthesize the oxides Co_3_O_4_,^41,42^ ZnMn_2_O_4_,^43^ PbO_2_,^44,45^ MnO_2_,^46–48^ and V_2_O_5_,^49^ as well as the trivalent oxyhydroxides NiOOH, CoOOH, FeOOH, and MnOOH.^32,50,51^

Different from the direct electrodeposition of metals and metal oxides, metal hydroxides can be deposited at the cathode *via* a two-step electroplating reaction. First, numerous OH^−^ ions near the surface of the cathode were generated *via* the reduction of solute. Then, metal ions in the solution are sedimented and deposited with OH^−^ in the form of a hydroxide. The key is to increase the surface pH of the working electrode. The following oxyacid anion reduction reactions are usually accepted to generate hydroxide ions:ClO_3_^−^ + 3H_2_O + 6e^−^ → Cl^−^ + 6OH^−^ *E*^θ^ = 1.890 VIO_3_^−^ + 3H_2_O + 6e^−^ → I^−^ + 6OH^−^ *E*^θ^ = 1.088 VNO_3_^−^ + H_2_O + 2e^−^ → NO_2_^−^ + 2OH *E*^θ^ = 0.838 V

For most metals, the standard potentials of the above three reactions are higher than those of the metal cation reduction. According to the Pourbaix diagram of nickel shown in Fig. 3C, one can choose the appropriate applied potential and pH of the electrolyte to control the types of deposits.^8^ When an aqueous solution of chlorate is used as the deposited electrolyte, hydroxyl groups will be generated *via* the reduction of chlorate and then increase the pH near the cathode surface. Nickel hydroxide is formed within the potential range of 0–1.23 V. This potential range can effectively avoid the negative influence from oxygen evolution (consumption of hydroxyl) and reduce Ni^2+^ to its metallic form. Similarly, for deposition electrolytes containing iodate or nitrate, nickel hydroxide will be produced in the applied potential ranges of 0–1.09 V and 0–0.84 V, respectively. Thus, the cathodic reduction increases the local pH value near the electrode and kinetically drives the deposition of metal hydroxides. In some cases, metal oxides will be formed when the hydroxide is further dehydrated or oxidized in air (Fig. 3D).^8,18^ Notably, cathodic electrosynthesis also favors the coprecipitation of bi-metallic or multi-metallic hydroxides. Cathodic electrodeposition is a general and efficient strategy to prepare hydroxides of different metals on a variety of conducting substrates. Up to now, almost all of the metal (hydro)oxides in the periodic table have been prepared *via* electrodeposition (Fig. 3E).

### Morphology design of metal (hydro)oxides

In recent years, nanostructured transition metal (hydro)oxides have attracted increasing attention due to their significant electrocatalysis properties. In addition to chemical composition, the catalysis efficiency and selectivity also rely on their shape, size, and even the separation distances of particles. Electrodeposition plays a vital role in producing a variety of nanostructured catalysts.^52–56^ In this section, we focus on the superiority of electrodeposition techniques in morphology design. Compared with other approaches, the synthetic variables of electrodeposition are easy to manipulate. In addition, electrodeposition not only allows precise control of the size, shape, composition and structure of the electrode, but also allows interfacial modification to improve the electrode stability. Accordingly, a variety of nanostructured (hydro)oxides with diverse morphologies have been prepared *via* electrodeposition, including low dimensional nanoparticles;^57,58^ core–shell,^59,60^ Janus,^61,62^ nanosheet and thin film structures;^63–67^ and three dimensional (3D) porous,^7,68,69^ nanowire,^70,71^ and nanotube array structures.^72,73^

#### Nanoparticle structures

Reducing the size of catalyst materials results in a significantly higher ratio of surface to bulk atoms, providing a relatively higher number of active sites. As a result, nanostructured materials often exhibit outstanding electrocatalytic performance compared with bulk materials. In principle, a synthetic method capable of producing fine grains can be used to produce nanomaterials. In the past few years, a number of important advances have been made in the electrodeposition of metal oxide nanoparticles such as NiO,^33^ Cu_2_O,^58^ CeO_2_,^8^ PbO_2_,^74^ IrO_2_,^75^ Co_3_O_4_,^76,77^ Fe_3_O_4_,^57^ ZnCo_2_O_4_.^43,78^

**Fig. 4:**
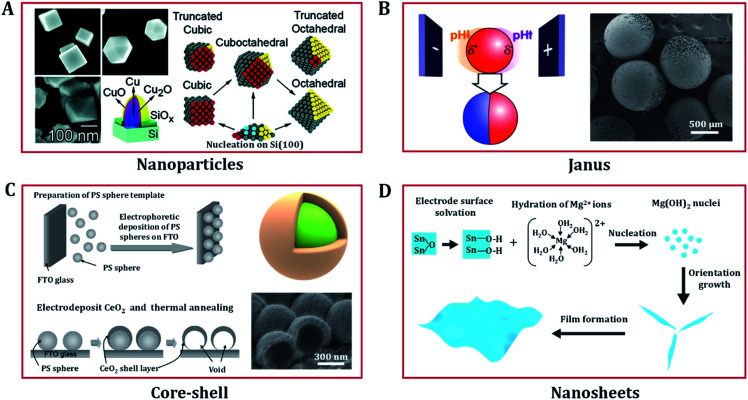
Low dimension nanostructured (hydro)oxides designed *via* electrodeposition. (A) Mechanism of the shape evolution of Cu nanoparticles and the SEM images of electrodeposited cubic, cuboctahedral, and octahedral Cu–Cu_2_O nanoparticles. Adapted with permission from ref. 58. Copyright 2010, the American Chemical Society. (B) Scheme of the indirect bipolar electrodeposition principle and an SEM image of Janus SiO_2_ carbon particles. Adapted with permission from ref. 62. Copyright 2012, the American Chemical Society. (C) Fabrication procedure of CeO_2_ core–shell nanostructures and FE-SEM images of CeO_2_ nanostructures electrodeposited on polystyrene (PS) sphere templates. Adapted with permission from ref. 59. Copyright 2009, the American Chemical Society. (D) Growth mechanism of the electrodeposition of porous nanostructures composed of nanosheets. Adapted with permission from ref. 67. Copyright 2012, the IOP Publishing.

Electrodeposition involves the deposition of ions on to an electrode surface in an electrolyte *via* electrochemical reactions using direct current or pulsed electric fields. In order to obtain nanomaterials, the nucleation rate needs to be increased and the growth rate decreased. Furthermore, the properties of the electrolyte (*e.g.* metal salt concentration and surfactant) can affect the morphologies, nanostructures, and orientations of the materials. As shown in Fig. 4A, Radi *et al.* obtained cubic, cuboctahedral, and octahedral Cu–Cu_2_O NPs with different average sizes by varying the deposition time and precisely controlling the electrolyte [CuSO_4_·5H_2_O] concentration at a constant potential of −1.0 V (with respect to a Ag/AgCl reference electrode).^58^

In addition to preparing common nanoparticles, electrodeposition can be used to fabricate electrodes with special structures, such as Janus and core shell nanoparticles. Janus particles exhibit different chemical characteristics on two opposite sides, making them a unique class of materials that present interesting properties, which have received strong scientific interest in the catalysis community. Kuhn's group proposed a straightforward bipolar electrochemical technology to produce sophisticated and highly composition-controlled Janus particles. By taking advantage of local pH change around the conducting particles to control the polymerization and/or precipitation of the insulating deposit, Janus particles of silica and crystalline titanium dioxide were successfully deposited on carbon or Pt particles (Fig. 4B).^61,62^ Core–shell nanostructured (hydro)oxides with a high surface area are also widely considered to be effective structures for applications in catalysts. Yamaguchi *et al.* prepared core–shell nanostructures of CeO_2_ through constant potential electrodeposition on a polystyrene sphere template (Fig. 4C).^59^ The structural morphologies of the deposit can be regulated by changing the Ce(NO_3_)_3_ concentration. Spherical CeO_2_ and needle-like Ce(OH)_3_/CeO_2_ shells are produced in 10 and 1 mM Ce(NO_3_)_3_ solutions, respectively.

#### Two-dimensional nanostructures

Benefiting from their large specific surface area and abundant edges, two-dimensional (2D) nanostructures can have abundant electrochemical active sites. Inorganic compounds with 2D nanostructures (such as single atom layers,^24^ nanosheets,^55,66,68^ and thin films^79,80^) are the key materials in various energy conversion devices. The fabrication of 2D compounds with high purity mostly relies on gas-phase methods such as chemical vapor deposition, vacuum evaporation, and sputtering. However, these routes usually require expensive equipment and complex energy-intensive processes. In contrast, bottom-up electrodeposition has distinct advantages in thin film materials preparation. As low processing temperatures minimize interdiffusion, uniform films can be deposited on diverse substrates with various different shapes. The film thickness can be precisely controlled by simply changing the delivered electrical charge. Thus, electrodeposition methods have been industrially used in surface protection, product decoration, metallic layer/foil plating, and anodization.

In addition to these traditional applications, electrochemical synthesis can also be used to produce advanced 2D nanoscale (hydro)oxide materials.^67^ For example, porous Mg(OH)_2_ thin films composed of single-crystal nanosheets can be easily obtained *via* cathodic electrodeposition. The growth mechanism of nanosheets is shown in Fig. 4D, and comprises four elementary stages: hydration, nucleation, growth, and thin film formation. OH^−^ ions play an important role in controlling the hydration rate of Mg^2+^ and the growth of Mg(OH)_2_ nuclei. In the nucleation process, the sp^3^d^2^ hybrid orbital of Mg^2+^ is empty and ready to accept electron pairs from OH^−^. The original nucleation of Mg(OH)_2_ on the electrode is rapid, creating a large mass of material, thus quickly blocking the electrode. The generated hydrogen bubbles randomly refresh some parts of the electrode and leave a number of nucleation sites for Mg(OH)_2_ nanosheets to form. The hydrogen bonding between charged and solvated surface groups promotes the formation of nanosheets. Similar electrodeposition routes have been used to grow NiO_*x*_,^81,82^ MnO_*x*_,^83^ ZnO_*x*_,^63^ CoO_*x*_,^84^ CeO_*x*_,^85^ CoNiO_*x*_,^86^ FeNiO_*x*_,^7,55^ CoFeO_*x*_ (ref. 68) and NiFeCuO_*x*_ (ref. 87) nanosheets. These films/foils/nanosheets have remarkable electrochemical properties for heterogeneous catalysis applications and beyond.^9,11,70–73^

#### Three-dimensional nanostructure

Traditional electrocatalysts in powder form must be integrated with conductive and binding agents to maintain good attachment and build pathways for charge transport. By combining multiple low dimension architecture materials, one can construct a continuous interconnected conductive 3D electrode. In particular, hollow 3D structures, which are beneficial for fast diffusion and enhanced OER kinetics, have attracted tremendous attention due to their abundant interior space and large surface areas. Electrodeposition is one of the most suitable methods for the preparation of 3D electrode materials. 3D (hydro)oxide nanostructures prepared *via* electrochemical synthesis can be divided into three types, *i.e.* hierarchical porous nanostructures (MnO_*x*_,^69^ Cu_2_O,^88^ Co_3_O_4_,^89^ NiFeO_*x*_H_*y*_^7^), nanowire arrays (CoFe_2_O_4_,^71^ MnO_2_ (ref. 70), and nanotube arrays (MnO_2_,^72^ CuO^90^). Most of these materials can be electrodeposited using template methods. Anodic aluminum oxide (AAO),^91–93^ ZnO nanorods,^48,94^ hydrogen/oxygen bubbles,^95–97^ and polystyrene^69,98^ have been widely used as templates. Electrodeposition ensures the uniform production of high-density 3D materials since it allows complete infilling of the space between the template spheres from the bottom to the top layers.

**Fig. 5:**
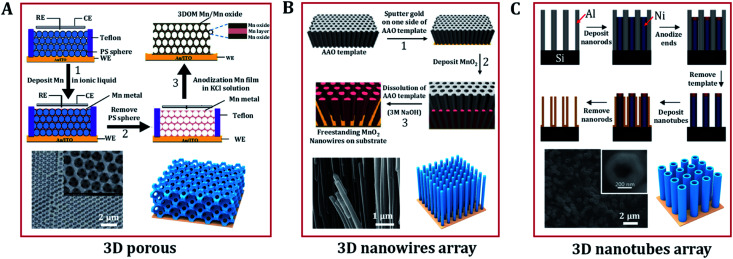
Typical 3D nanostructures of (hydro)oxides designed by electrodeposition. (A) Scheme of the preparation of a high-porosity 3D ordered macroporous Mn/Mn oxide electrode and its related SEM image. Adapted with permission from ref. 69. Copyright 2013, the Royal Society of Chemistry. (B) Schematic diagram representing the electrochemical template synthesis of MnO_2_ nanowires arrays and SEM micrographs of manganese oxide nanowire arrays. Adapted with permission from ref. 53 and 70. Copyright 2013, the American Chemical Society and Copyright 2003, the Elsevier B.V. (C) Schematic illustration of the fabrication of nanotube arrays on substrates and SEM images of MnO_2_/PPy/MnO_2_ nanotubes arrays. Adapted with permission from ref. 48 and 73. Copyright 2005, the American Chemical Society.

Polystyrene has been widely used as template because it has the advantage of being easily removed by simple dissolution in tetrahydrofuran or high temperature calcination without causing any damage to the target porous architecture. Moreover, the natural abundance and low cost of Mn oxides, and their satisfactory electrochemical performance have made them promising electrode materials for the OER. Deng *et al.* prepared a 3D ordered microporous Mn core–MnO_2_ shell structure *via* the electrodeposition of metallic Mn within a polystyrene template in an ionic liquid (Fig. 5A).^69^ The 3D Mn/MnO_2_ structure provides good ionic conduction in the electrolyte, which is favorable for catalysis under a large current density. Miyake *et al.* demonstrated that high-quality 3D Cu_2_O crystals could also be created *via* electrodeposition in a polystyrene template.^88^ The contiguous internal space of the polymer template was completely filled with crystalline Cu_2_O through bottom-up growth *via* electrodeposition. Then, etching of the template was carried out to make the Cu_2_O crystals have the exact inverse structure of the template. This approach also enables good control over the diameter and length of nanowires. Duay *et al.* explored a simple template-assisted anodic deposition process to produce nanowire arrays of MnO_2_ (Fig. 5B).^53^ This route does not require the use of any adhesives or conductive additives because the independent MnO_2_ nanowires are directly connected to a gold current collector. This is beneficial to the exposure and utilization of catalytic active sites.

Due to the advantages of a large specific surface area, high conductivity, and good chemical stability, nanotube arrays also show great potential in the catalytic reaction of oxygen evolution. Sander's group designed a double-template approach to create aligned arrays of nanotubes on substrates,^73^ shown schematically in Fig. 5C. Initially, nanoporous templates were fabricated by anodizing aluminum films that were evaporated onto silicon substrates. Parameters such as the pore diameter, height, and pore ordering can be controlled by changing the anodization conditions. Next, nickel nanorods were electrodeposited into the pores of the alumina. After deposition, the exposed ends of the nanorods were modified *via* anodization to prevent further deposition. The alumina template was then removed *via* selective chemical etching, leaving an array of nickel nanorods upon which to electrodeposit metal or (hydro)oxide nanotube arrays. Except for the anodized tips of the nanorods, the nanotube material deposits uniformly across the entire surface of the nanorod arrays. Finally, through the selective removal of the nickel nanorod array template, arrays of open-ended nanotubes were thus formed in the substrate. Many oxide (CuO,^99^ Fe_2_O_3_,^100^ ZnO,^101^ MnO_2_,^72^ V_2_O_5_ (ref. 49)) nanotube arrays have been created using a similar approach.

In principle, electrodes and materials with tailored structures can are accessible using simple electrochemical procedures. If deposited in aqueous solutions, the procedure is limited to elements that are more noble than hydrogen. For the deposition of active metal-based materials, the procedure can be achieved in ionic liquids, organic solution or even molten salts.^27,28,40,102^ In particular, directly fabricating nanostructured (hydro)oxides with a selective morphology and high surface area on to a conductive substrate using electrodeposition routes has become the focus of recent research. These unique nanostructured (hydro)oxides are expected to be promising functional electrodes materials for OER catalytic applications.

### Applications of (hydro)oxides in the OER

As an established technique, the electrochemical splitting of water has been known since the 19th century. However, the anodic reaction of water oxidation remains a mystery. The accurate reaction mechanism has not yet been fully understood and the ideal catalyst is still under development, leaving numerous unsolved problems and big challenges in the OER field. From an energy and environmental crisis perspective, it is extremely urgent to develop high activity, stable and inexpensive OER electrocatalysts. It is generally accepted that optimizing the design of catalysts requires a better understanding of electrochemical reaction mechanisms. Herein, we aim to summarize the recent development in the fundamental understanding of OER related to metal (hydro)oxide catalysts. Then, we enumerate the benchmark electrocatalysts prepared *via* electrodeposition.

**Fig. 6:**
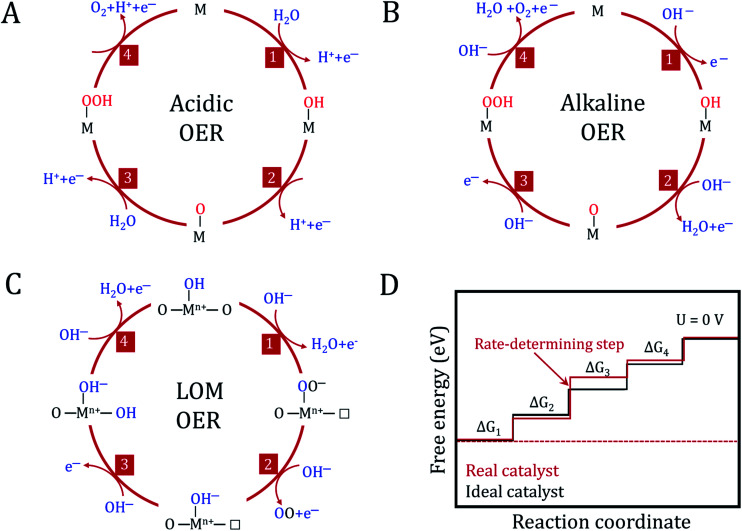
The oxygen evolution reaction mechanism. Conventional OER mechanism for acidic (A) and alkaline (B) conditions. (C) Reaction mechanism for the OER, including the participation of lattice oxygen in an alkaline environment. M represents the active site and □ represents the oxygen vacancy. (D) Plot of the Gibbs free energy of reactive species and intermediates (horizontal lines) of the OER *versus* the reaction coordinates. The blue and red lines indicate the energetics of a real and ideal catalyst, respectively. The dashed lines indicate the energetics at the electrode potential where all of the thermochemical barriers disappear.

In the mechanism of the OER on metal (hydro)oxides, it is traditionally considered that the reaction overpotential is mainly governed by the binding strength of O-containing intermediates on the catalyst surfaces, which is also identified as an adsorbate evolving mechanism (AEM).^3^ For an ideal catalyst, the absorbed O-containing species on its surface should bind neither too strongly nor too weakly, as described in the Sabatier principle.^103^ To understand the detailed OER process, several reaction mechanisms regarding the metal centers as active sites have been proposed in the past few decades. As shown in Fig. 6A and B, the conventional OER mechanism of (hydro)oxides from previous experimental and computational studies involves four consecutive proton-coupled electron transfer steps. These processes occur on single metal or adjacent metal sites.^104–106^

The detailed mechanisms of the OER in acidic and basic electrolyte are as follows.

OER in acidic electrolyte:* + H_2_O → OH* + H^+^ + e^−^OH* → O* + H^+^ + e^−^O* + H_2_O→ OOH* + H^+^ + e^−^OOH* →* + O_2_ + H^+^ + e^−^

OER in alkaline electrolyte:* + OH^−^ → OH* + e^−^OH* + OH^−^ → O* + H_2_O + e^−^O* + OH^−^ → OOH* + e^−^OOH* + OH^−^ → * + O_2_ + H_2_O + e^−^where * denotes the active site of the catalyst, and OH*, O*, and OOH* represent adsorbed intermediates species. The change in the adsorption Gibbs free energy (Δ*G*) of each oxygen species is different in real catalysts.

In addition to these two conventional mechanisms, the one with lattice oxygen (O^2−^) catalyst participation has recently been extensively considered as an alternative reaction pathway in (hydro)oxides (Fig. 6C).^107^ The Sabatier principle is still valid in the lattice oxygen mechanism (LOM) process, but it needs to noted that the active sites are no longer limited to the metal centers. Dynamic catalyst active sites may also arise as a result of the oxidation of O^2−^ anions. Undoubtedly, it becomes more complex to determine a single guiding parameter in the LOM to describe OER activity.

Having a detailed understanding of the interaction between oxygen intermediates and the catalyst surface is important for the improvement in the overall OER performance. However, a thorough understanding of each elementary step and confirming the kinetic descriptions of the OER is very difficult in practice. Fortunately, density functional theory (DFT) calculations provide a simple route and deep insight into the individual reaction steps. Extensive attention has been paid toward modeling the thermodynamics of electrochemical reactions of (hydro)oxides for the OER process. The DFT pioneer Nørskov *et al.* established a universal framework for OER kinetics on metal (hydro)oxide surfaces.^3,108^ Typically, the theoretical OER overpotential (*η*) among different catalysts can be correlated to a single descriptor following the Sabatier principle. In particular, the reaction energy of each elementary step is determined by the difference in the adsorption energy between two intermediates (*e.g.* Δ*G*_O*_ − Δ*G*_OH*_), and the reaction free energy diagrams are drawn to determine the thermodynamically rate-determining step, as shown in Fig. 6D. The reaction energies for each step are different owing to the irregular variations in the adsorption energies of the intermediate species. The step with maximum free energy is the rate-determining step, which is responsible for the *η* for the OER. In an ideal catalyst, the free energy of each step is equal to achieving a minimum overpotential. To minimize the overpotential, the binding energy of the intermediates can be tuned according to the metal type, electronic structure, adsorbed species, and solvent interactions, *etc.*

**Fig. 7:**
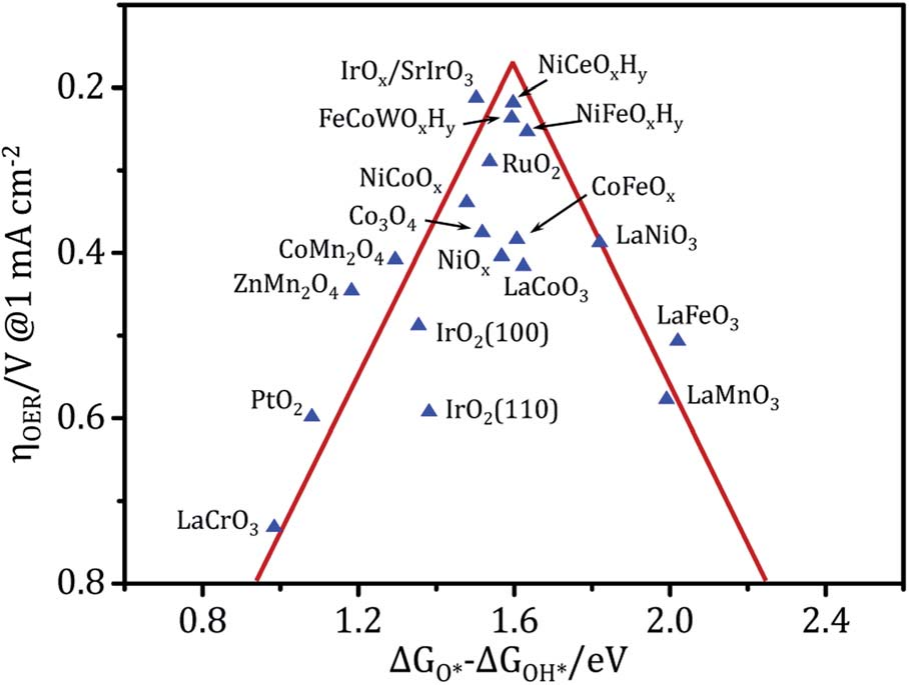
Activity trends towards the oxygen evolution of (hydro)oxides. The OER overpotential data in the vertical coordinates was measured experimentally and the adsorption energy between two intermediates (Δ*G*_O*_ − Δ*G*_OH*_) in the abscissa is the theoretical data calculated using DFT, where all of the data were obtained from the literature.

Owing to the high cost and scarcity of precious metal oxides such as RuO_2_ and IrO_2_, non-precious metal (hydro)oxide catalysts such as nickel, iron, cobalt, manganese, and multi-metal based (hydro)oxides have been vigorously studied. The metal (hydro)oxides can be mainly classified into three categories: NiCeO_*x*_H_*y*_,^8,109^ FeCoWO_*x*_,^110^ CoFeO_*x*_,^111^ FeNiO_*x*_,^7^ and CoFeNiO_*x*_ (ref. 32) belong to the first group, which is the most active; CoO_*x*_ (ref. 41) and NiCoO_*x*_ (ref. 112) belong to the second group; and FeO_*x*_, MnO_*x*_, LaCrO_*x*_, LaMnO_*x*_ and LaFeO_*x*_ belong to the third group,^105,113^ which only has modest activity even at relatively high overpotentials. Fig. 7 shows the relationship between the experimental overpotential at 1 mA cm^−2^ and the free energy difference of Δ*G*_O*_ − Δ*G*_OH*_ in metal (hydro)oxides. The results reveal that these (hydro)oxides display a volcano-shaped relationship and that the highest activity corresponds to an intermediate binding strength of *ca.* 1.6 eV.^3,110,114^ The materials at the top of the volcano have been experimentally proven to be among the best OER catalysts to date under alkaline conditions. However, they are still far from ideal in terms of activity and stability under a large current density. Benefiting from its strong controllability and universality, electrodeposition holds tremendous potential for preparing electrocatalysts for water splitting. Advanced (hydro)oxide OER electrocatalysts with good electrocatalytic activity can be obtained *via* electrodeposition. In particular, electrodeposition usually combines interface modification and 3D porous structure construction. This produces electrodeposited electrodes with a large active surface area, and high speed mass transport and fast electron transport properties. These electrodeposited electrodes not only have ultra-high activity, but also excellent stability under high current density catalysis.

Bose *et al.* reported an efficient CeO_*x*_/NiFe–OH composite oxygen evolution electrocatalyst, which was electrochemically deposited on a nickel foam substrate.^35^ The synthesized electrocatalyst exhibited good OER performance, reaching a current density of 100 mA cm^−2^ at a low overpotential of 280 mV. This is superior to most of the previously reported non-noble metal-based OER electrocatalysts. Hu's group developed an oxidative electrochemical method to deposit a family of thin-film transition metal layered hydroxides as OER catalysts.^32^ The electrodeposition method allowed precise control and measuring of the catalyst loading using an electrochemical quartz crystal microbalance. The precise catalyst loading made it possible to test the intrinsic activity of the turnover frequencies. The CoFeNiO_*x*_ electrode with high turnover frequency required overpotentials of as low as 270 mV to reach a current density of 100 mA cm^−2^ in 1.0 M KOH. Zhao's group explored an efficient oxygen electrode by electrodepositing amorphous mesoporous nickel-iron composite nanosheets directly onto nickel foam substrates.^7^ The as-prepared electrode can deliver a current density of 1000 mA cm^−2^ towards water oxidation at an overpotential of 270 mV in alkaline solutions. Our group further developed the universal electrosynthesis of NiCeO_*x*_H_*y*_ on graphitic substrates. The insertion of nitrate ions in graphene layers significantly enhanced the electrodeposit-support interface, resulting in a superhydrophilic electrode with high mass loading.^8^ The self-standing electrode exhibits a low overpotential and long-term durability (over 300 h) at a high current density of 1000 mA cm^−2^. Benefiting from the modification of the electrode interface, the oxygen electrode can even withstand a current density of 5000 mA cm^−2^. Such advances have certain guided significance for the interface-controllable electrosynthesis of advanced electrodes viable to industrial applications. Thus, electrodeposition provides a promising inexpensive, yet efficient way to prepare catalysts for industrial electrolyzers.

## Conclusions and outlook

Electrodeposition is a simple and versatile technology that can be used to prepare a variety of (hydro)oxide materials with different nanostructures for OER applications. It is drawing increasing attention from materials scientists and chemists in both fundamental and applied research. Thus, the properties of (hydro)oxides, the design strategies of electrodeposition nanostructures, OER mechanisms, and benchmark (hydro)oxide catalysts from over the past few decades have been summarized. As highlighted, a variety of nanostructured materials of various sizes, morphologies, dimensions, and compositions can be precisely fabricated for advanced OER electrode applications *via* adjusting the synthetic parameters. In particular, transition or lanthanide oxide modified hydroxide composite electrodes, *e.g.* NiCeO_*x*_H_*y*_, which can be prepared *via* electrodeposition will bring about more possibilities for water splitting.

Benefiting from it strong controllability and universality, electrodeposition technology is particularly suitable for preparing electrodes for the OER used in water splitting devices. Continuous research and development should focus on the deeper understanding of the underlying mechanisms to make this strategy more convincing and versatile. The deposition process and the individual behavior of many different structured nanoparticles, such as *in situ* visualization and quantification of electrochemical Ostwald ripening, require *in situ* and real time monitoring. We think that a quick shift in research attention from simple and direct use to an in-depth understanding of the fundamental principles of electrodeposition would stimulate the full potential of this technology. In addition, it has great potential to be used to controllably synthesize newly discovered functional materials such as conductive MOFs and single atom materials.^115^ Although electrodeposition is known as a well-developed and widely-applied technique, current schemes still have intrinsic limitations and unsolved difficulties. For OER electrocatalysts, free energies of adsorption are the key to bridging experimental and computational results. Identifying active sites and reaction intermediates through operando technology will greatly promote mechanism understanding and materials design. Overall, we believe that the (hydro)oxide electrodeposition avenue discussed here could provide exciting opportunities for the practical application of electrolytic water technology.

## Conflicts of interest

There are no conflicts to declare.
